# Impact of JNK and Its Substrates on Dendritic Spine Morphology

**DOI:** 10.3390/cells9020440

**Published:** 2020-02-14

**Authors:** Emilia Komulainen, Artemis Varidaki, Natalia Kulesskaya, Hasan Mohammad, Christel Sourander, Heikki Rauvala, Eleanor T. Coffey

**Affiliations:** 1 Turku Bioscience, University of Turku and Åbo Akademi University, Tykistokatu 6, 20500 Turku, Finland; H.E.Komulainen@sussex.ac.uk (E.K.); artemisstef@gmail.com (A.V.); hasanjogi@gmail.com (H.M.); christel.c.sourander@utu.fi (C.S.); 2 University of Helsinki, Neuroscience Center, 00014 Helsinki, Finland; natalia.kulesskaya@helsinki.fi (N.K.); heikki.rauvala@helsinki.fi (H.R.)

**Keywords:** JNK1, JNK, synaptic plasticity, dendritic spine, substrate, hippocampus, DCX, MARCKSL1, MRP, Morris water maze

## Abstract

The protein kinase JNK1 exhibits high activity in the developing brain, where it regulates dendrite morphology through the phosphorylation of cytoskeletal regulatory proteins. JNK1 also phosphorylates dendritic spine proteins, and *Jnk1-/-* mice display a long-term depression deficit. Whether JNK1 or other JNKs regulate spine morphology is thus of interest. Here, we characterize dendritic spine morphology in hippocampus of mice lacking *Jnk1-/-* using Lucifer yellow labelling. We find that mushroom spines decrease and thin spines increase in apical dendrites of CA3 pyramidal neurons with no spine changes in basal dendrites or in CA1. Consistent with this spine deficit, *Jnk1-/-* mice display impaired acquisition learning in the Morris water maze. In hippocampal cultures, we show that cytosolic but not nuclear JNK, regulates spine morphology and expression of phosphomimicry variants of JNK substrates doublecortin (DCX) or myristoylated alanine-rich C kinase substrate-like protein-1 (MARCKSL1), rescue mushroom, thin, and stubby spines differentially. These data suggest that physiologically active JNK controls the equilibrium between mushroom, thin, and stubby spines via phosphorylation of distinct substrates.

## 1. Introduction

The hippocampus is the most extensively studied structure with regards to memory and learning. Changes in dendritic spine density and shape in the hippocampus correlate with spatial and associative learning in rodents [1,2,3,4]. Increased spine density improves synaptic efficacy [5] and increased spine head size correlates with synaptic strength [6,7,8]. Such strengthening of synaptic currents, achieved through growth and stabilization of synapses, is known as long-term potentiation (LTP). Weakening of synaptic currents on the other hand, achieved through spine shrinkage, is known as long-term depression (LTD). Both forms of synaptic plasticity contribute to information storage and spatial memory formation in the hippocampus [9,10,11,12]. Moreover, loss of synapses in specific brain regions correlates with depression severity and maladaptive circuit activities in patients with major depression or post-traumatic stress disorder [13]. Similar synapse loss is observed in animal models of depression [14,15]. Understanding the molecular mechanisms that control spine morphology and density in the nervous system is therefore of broad interest, as this underlies basic neurophysiology and brain function.

In the mouse nervous system, three *Jnk* genes are expressed, *Jnk1*, *Jnk2*, and *Jnk3* [16]. Knockout studies have established that JNK isoforms contribute differentially to synaptic plasticity. Thus, *Jnk1* is required for metabotropic glutamate receptor (mGluR)-dependent LTD in young mice but not for LTP [17], whereas *Jnk2* is required for hippocampal LTP [18,19]. Use of a pan-JNK inhibitor (SP600125) that does not distinguish between isoforms prevents amyloid-beta peptide-induced LTD [20] and LTP [21,22], whereas beta-amyloid-induced neuron death is associated with JNK3 and may be relevant for Alzheimer’s disease [23,24]. Finally, *Jnk1-/-* mice show increased baseline contextual fear responses, suggesting that JNK1 negatively regulates this form of association memory [19]. Thus JNK isoforms have been associated with various forms of synaptic plasticity, yet less is known regarding the underlying mechanisms.

Among the phosphorylation targets of JNK1 are regulators of microtubule and actin dynamics. JNK1 regulates neuronal architecture and neuronal migration by phosphorylating targets such as microtubule-associated protein 2 (MAP2), stathmin-2, myristoylated alanine-rich C kinase substrate-like protein-1 (MARCKSL1), and doublecortin (DCX) [25,26,27,28,29,30,31]. Studies in *Jnk1-/-* mice demonstrated that JNK1 controls dendritic arborisation in the cortex and cerebellum [26,30]. Use of an optogenetic JNK inhibitor revealed that JNK activation within dendritic spines triggers spine regression in response to NMDA or glucocorticoid stress [31]. Moreover, this study showed that JNK controls actin dynamics in thin spine heads. These studies suggest that JNK isoforms regulate structural and neurophysiological aspects of synaptic plasticity. However, information on dendritic spine structure from *Jnk* knockout mice is lacking, as is the role of JNK substrates in spine morphology regulation.

Here, we examined the dendritic spine structure in mice lacking *Jnk1*, the physiologically active JNK in the brain [32,33]. We found that spine maturation and density was significantly altered and spatial memory was impaired. We also examined the role of JNK1 targets DCX and MARCKSL1 in determining spine structure.

## 2. Materials and Methods

### 2.1. Animals

*Jnk1-/-* (B6.129S1-Mapk8tm1Flv/J) or wild-type mice (C57B6/J) were used. For behavioural testing, 8 × *Jnk1-/-* and 10 × wild-type littermate mice (all male) were used. Animals were group-housed (4–5 animals per cage) in standard cages with bedding and nesting material under a 12 h light–dark cycle. Food and water were provided ad libitum. Behaviour assessment was started when animals were 8–9 weeks old. Behaviour experiments were performed with 1–2 day intervals between tests. Tests were carried out between 10:00 and 15:00. Animal procedures were approved by the Animal Experiment Board in Finland. For brain homogenates from *Jnk1-/-*, *Jnk2-/-*, or *Jnk3-/-* mice, the animals were described previously [16,34].

### 2.2. Morris Water Maze

The water maze consisted of a black round tank (diameter 120 cm) filled with clean room temperature water that was divided virtually by four equal quadrants. A black escape platform (diameter 10 cm) was submerged 0.5 cm under the water surface and placed in the middle of one of the quadrants. The test lasted 6 days and consisted of (i) learning acquisition of the hidden platform (day 1–3: three trials per session, two sessions per day, six sessions in total) and the following day, a probe trial without platform (day 4: one trial, 60 min), (ii) “reversal” learning of the “new” hidden platform located in the opposite quadrant to the “old” platform (day 4–5: three trials per session, two sessions per day, four sessions in total) and a probe trial without a platform (day 6: one trial, 60 sec), and (iii) one session with a visible platform located in a new place to test motivation and vision (day 6: three trials). The inter-trial latency was about 5 min, and the interval between the sessions on the same day was about 4–5 h. The mouse was released to swim in different locations of the maze facing the wall, and was video-tracked using the Noldus EthoVision 8.0 system (Noldus Information Technology, Wageningen, The Netherlands) for 60 sec or until it escaped to the platform. The latency and the distance to escape to the hidden platform and the time spent in each quadrant was analysed automatically for learning sessions and for probe trials, respectively.

### 2.3. Plasmids

CAG–GFP, pEGFP-NES-JBD, and pEGFP-NLS-JBD have been previously described [26]. pEGFP-MARCKSL1-S120D/T148D/T183D (MARCKSL1-DDD) has also been described previously [29]. mRuby-LifeAct was from Michael Davidson [35]. pEGFP-DCX S334 and Thr331, alanine, and aspartate mutants were generated using site-directed mutagenesis using the following primers: forward: 5′ GTCTCCCATCTCTGATCCCACCGATCCTGGCAGCCTCC 3′ reverse: 5′ GGAGGCTGCCAGGATCGGTGGGATCAGAGATGGGAGAC 3′ as previously described [36]. For insertion into pEGFP C2 vector (Clontech, Mountain View, CA), the following primers were used: forward 5′ attcttaGAATTCATGGAACTTGATTTTGGACACT 3′ reverse: attcttaCTCGAGTTACATGGAATCACCAAGCG. Digestion was performed with EcoRI (Promega) and XhoI (Promega) restriction enzymes. EGFP-DCX was a gift from Joseph Gleeson (Addgene plasmid #32852) [37]. mU6pro-DCX shRNA (3′UTRhp) and a mutant shRNA (mU6pro-Dcx shRNA M3) were from Joseph Loturco [38]. pCMV empty vector was used to make up the final concentration during transfections.

### 2.4. Antibodies

Phospho-DCX antibody P-DCX-334 (#3453) was from Cell Signalling Technology (Leiden, The Netherlands). Antibodies P-DCX-1952 (T331, S334) and P-DCX-1774 (T321, S327) were gifts from Orly Reiner (Weizmann Institute, Rehovot, Israel). DCX antibody (#271390) was from Santa Cruz Biotechnology (Dallas, Texas, USA). Antibodies against JNK1 and JNK3 have been previously described [39].

### 2.5. Cell Culture and Transfection

For cortical neurons, cortices were collected from new-born (P0) C57Bl/6 wild-type or *Jnk1-/-* mice, and the primary cell culture was prepared as previously described [26]. For hippocampal cultures, newly born (P0) Sprague-Dawley pups were used as previously described [40]. For both culture types, cells were maintained in Neurobasal A supplemented with B27 and penicillin/streptomycin (concentration). Transfections were performed when neurons were 7 days in vitro (DIV) using Lipofectamine LTX (Invitrogen, Carlsbad, CA, USA). The medium was changed to Neurobasal A before transfection. A total of 0.5 µg plasmid DNA (as indicated) was mixed with 0.125 µL PLUS reagent for 10 min, and then combined with 1 µL LTX for 30 min before adding to cells. After 40 min incubation, conditioned medium was returned. The cells were maintained for 21 days in a humidified incubator with 5% CO_2_ at 37 °C and were fixed using 4% paraformaldehyde. Coverslips were mounted on slides using mounting medium made from 2.4 g of Mowiol 4–88 in 6 g glycerol containing 2.5% 1,4-diazabicyclo[2.2.2]octane (DABCO) (Sigma-Aldrich).

### 2.6. Mouse Perfusion and Lucifer Yellow Loading of Cells

Mice at 6 to 10 months were anesthetised with 0.3 mg/g Pentobarbital (Mebunat 60 mg/mL) mixed 50:50 with 0.9% NaCl. Mice were perfused transcardially on ice bedding using 10–20 mL 0.9% NaCl followed by 25–50 mL of buffer comprising 4% paraformaldehyde and 0.125% glutaraldehyde in 0.1 M Sorensen’s phosphate buffer (NaH_2_PO_4_-Na_2_HPO_4_, pH 7.2). The brain was post-fixed in 50 mL 4% paraformaldehyde in phosphate buffer for 4–12 h at +4 °C. Coronal sections of 180 to 200 µm were sectioned using a vibratome. The nuclei were visualized using DAPI (4′,6-diamino-2-phenylindole, dihydrochloride; InvitrogenSelected)). Neurons were injected by iontophoresis with Lucifer yellow dye (Invitrogen) using pulled borosilicate glass tubes (World Precision Instruments). The DC current source was 2–6 nA from a dual micro-iontophoresis current generator, model 260 (World Precision Instruments). After dye loading, brain slices were transferred to a slide and mounted using Shandon PermaFluor mounting medium (ThermoFisher).

### 2.7. Spine Imaging and Analysis: In Vivo

For Z-stacks of dendrites from Lucifer yellow-loaded neurons, images were acquired from the second and third order dendrites 25–75 µm from the soma, along a dendritic length of 25 µm using a 100x objective with numerical aperture of 1.40 with a Leica TCS SP1 microscope, and a z-section thickness of 0.203 µm. Apical and basal dendrite spines were analysed. For *in vitro* imaging of EGFP-expressing cortical or hippocampal neurons in culture (as indicated), imaging was performed using the Zeiss LSM-780 with a z distance of 0.378 μm. Images were acquired from dendrites within a radial distance of 25 μm from the soma using a Plan-Apochromat 63×/1.4 Oil DIC objective. Apical and basal dendrites could be distinguished in most cells from these primary cultures. Only cells with a clear apical/basal appearance were used. For both in vivo and in vitro spines, images were deconvoluted using Autoquant X3 software as part of the Imaris imaging package (30 iterations). Spine segmentation was performed with Imaris 8.3 using Filament Tracer and Classify Spine XTension modules (Bitplane AG, Zurich, Switzerland). Spines were manually counted from Z-stacks using Neurolucida Explorer (MBF Bioscience, Williston, DC, USA) and categorised manually on the basis of morphology into mushroom (large bulbous head with a neck constriction), stubby (no neck constriction), and thin (long neck and small head) forms. Imaging of slides and analysis of images were performed by a blinded experimenter. For spine morphology analysis, mushroom spines were defined as those with a head/neck ratio >2; thin spines had a head/neck ratio <2. Spines with no neck that were attached to the dendritic shaft were defined as stubby, as has been done previously [41,42].

### 2.8. Statistical Analysis

For Morris water maze (MWM) behaviour testing, repeated two-way ANOVA was used to determine *p*-values over the entire training period followed by post-hoc Sidak test. Adjusted *p*-values are shown in the graphs. Student’s *t*-test was used where indicated to determine *p*-values between genotypes for individual quadrants. For analysis of Lucifer yellow-loaded neurons, Student’s *t*-test was used. For spine analysis, one-way ANOVA with Sidak test for multiple comparisons was used. Adjusted *p*-values are shown.

## 3. Results

### 3.1. Spatial Learning of the Jnk1-/- Mice Was Compromised

**Figure 1:**
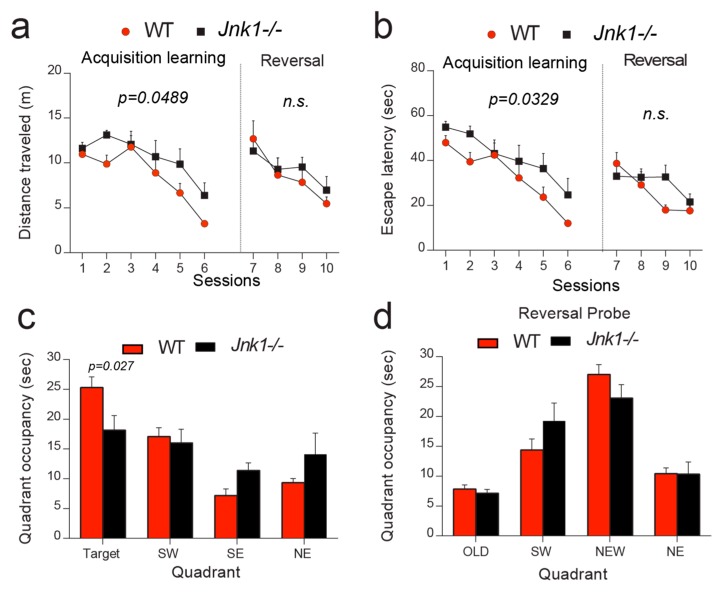
*Jnk1-/-* mice displayed learning and memory deficits in the Morris water maze. (**a**,**b**) During the acquisition phase, *Jnk1-/-* mice performed poorly compared to wild type traveling for a longer time (**a**) and showed increased latency to learn (**b**). *p*-values were calculated from ANOVA with repetition followed by post-hoc Sidak test. In the reversal phase, *Jnk1-/-* mice did not differ from wild-type mice. (**c**). In the probe test, *Jnk1-/-* mice spent significantly less time swimming in the target quadrant (*p* = 0.027, Student’s *t*-test) and showed no preference for target quadrant. However wild-type mice spent more time in the target quadrant than the southwest (SW) quadrant (*p* = 0.0071), the southeast (SE) quadrant (*p* < 0.0001), or the right northeast (NE) quadrant (*p* < 0.0001). *p*-values were calculated using ANOVA with Dunnett’s post-hoc test. (**d**). There was no genotype difference in reversal learning.

To test if spatial learning and memory was affected in *Jnk1-/-* mice, animals were subjected to the Morris water maze (MWM) test [43]. This consisted of three training sessions per day for 6 days, followed by a probe test without platform. A well trained mouse will spend close to 50% of the time scanning the original target quadrant [44]. We found that without a difference in swimming speed, *Jnk1-/-* mice travelled a greater distance and spent more time trying to find the hidden platform during acquisition learning, performing worse than wild-type mice (Figure 1a,b). In addition, in the probe trial without a platform, *Jnk1-/-* mice spent less time in the target quadrant compared to wild-type mice (Figure 1c). However, during the reversal phase, when mice relearned a new place for the hidden platform, there was no genotypic difference (Figure 1a,b), and *Jnk1-/-* and wild-type mice both spent most of the time swimming in the “new target” quadrant during the reversal probe trial (Figure 1d). As both genotypes performed at an equal level when the platform was visible, visual acuity or motivation was not compromised in knockout mice. *Jnk1-/-* mice struggled only in the first navigation task with a hidden platform, implying impaired spatial learning and memory.

### 3.2. In Jnk1-/- CA3, the Percent of Mushroom Spines Was Decreased and Thin Spines Increased

**Figure 2:**
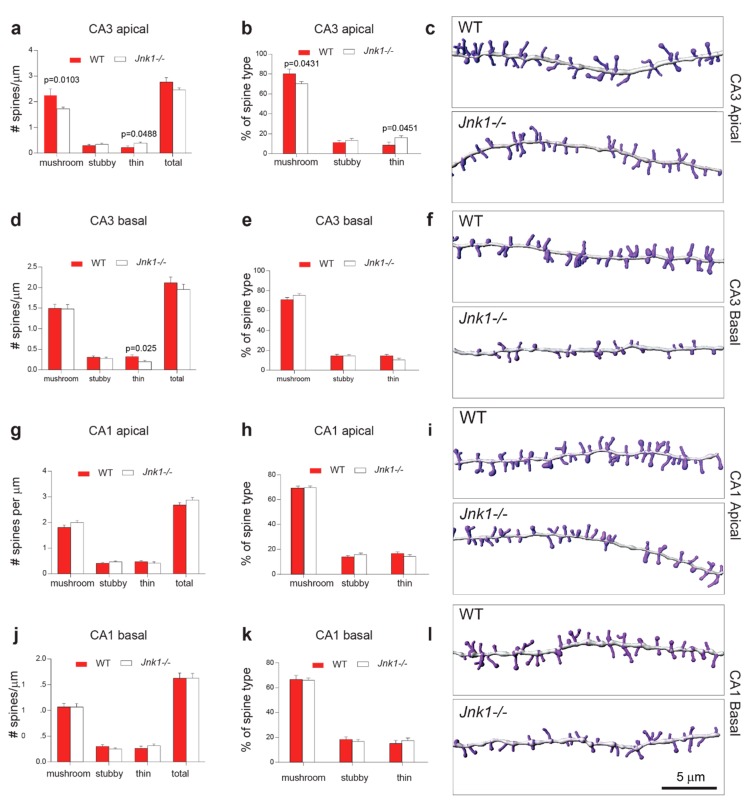
*Jnk1-/-* mice showed loss of mushroom spines and increased immature spines in hippocampus CA3. Spine density and spine morphology data obtained from Lucifer yellow labelling of second and third order dendrites in wild-type and *Jnk1-/-* hippocampus CA3 of adult mice. Spine measurements were taken from 25 to 30 µm segments in the shaft. Apical and basal dendrites were measured. Spines were manually categorized as mushroom, stubby, or thin morphology using Neurolucida software. (**a**,**b**) *Jnk1-/-* CA3 pyramidal neurons showed decreased mushroom spine number. Number of CA3 apical dendrites measured: Wild-type (WT) *n* = 5, *Jnk1-/- n* = 16. (**c**). For representation purposes, dendrites and spines were segmented using Imaris software. (**d**–**f**). The same is shown for basal dendrites in CA3 and (**g**–**i**) apical and basal (**j**–**l**) spines in CA1. Number of basal dendrites measured in CA3: WT *n* = 17, *Jnk1-/- n* = 24; number of apical dendrites in CA1: WT *n* = 19, *Jnk1-/- n* = 15; number of basal dendrites in CA1: WT *n* = 14, *Jnk1-/- n* = 21. No significant changes were observed in hippocampus CA1. *p*-values were calculated using Student’s *t*-test.

As spatial learning is associated with synaptic changes in the hippocampus, we used Lucifer yellow labelling to examine spine density and morphology in CA3 and CA1 hippocampal subdomains from male wild-type and *Jnk1-/-* mice at 6 to 10 months old. Dendrites from dye-loaded neurons were imaged using confocal microscopy, and three-dimensional reconstructions were analysed using Neurolucida software. The proportion of mushroom spines was decreased and that of thin spines increased in apical dendrites of hippocampus CA3 from *Jnk1-/-* mice (Figure 2a–c). Specifically, mushroom spine density was reduced compared to wild-type mice at 2.25 ± 0.25 spines/µm to *Jnk1-/-* mice at 1.72 ± 0.07 spines/µm, and there were 10% fewer mushroom spines overall. For thin spines, density increased from 0.22 ± 0.09 spines/µm in wild-type mice to 0.39 ± 0.09 spines/µm in *Jnk1-/-* mice, representing a doubling of thin spines in *Jnk1-/-* mice (Figure 2a).

In the basal dendrites of hippocampal CA3, there was a modest but significant decrease in density of thin spines (wild-type 0.31 ± 0.04 spines/µm, *Jnk1-/-* 0.2 ± 0.39 spines/µm) (Figure 2d–f), whereas mushroom spines were unchanged. In hippocampal CA1, there was no genotypic difference in spine density between wild-type and *Jnk1-/-* brains, either for apical or basal dendrites (Figure 2g–l).

### 3.3. Mushroom Spines Were Decreased in Both Apical and Basal Dendrites in Jnk1-/- Neurons

**Figure 3:**
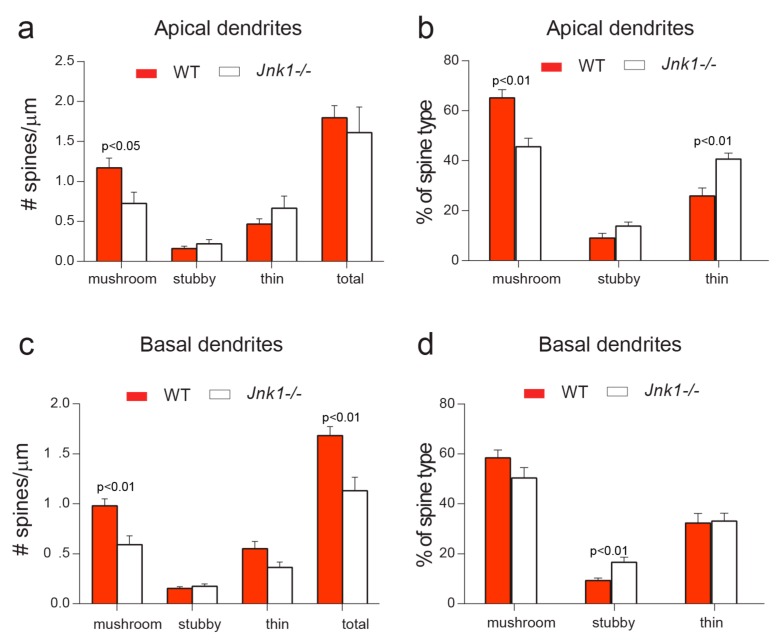
Spine density was altered in cortical neurons from *Jnk1-/-* mice in culture. Cortical neurons were transfected with EGFP and spines were measured when cells were 20 days post-plating. (**a**,**b**) Spines were manually categorized into mushroom, thin, or stubby. Mushroom spines were decreased in apical dendrites from *Jnk1-/-* neurons, whereas thin spines were increased. For apical dendrites in cortical neurons: WT *n* = 8, *Jnk1-/- n* = 8. (**c**,**d**). For basal dendrites, mushroom spines were decreased, as was total spine density. Additionally, the proportion of stubby spines increased. WT *n* = 11, *Jnk1-/- n* = 10. *p*-values were calculated using Student’s *t*-test.

These results prompted us to examine spine characteristics from cortical neurons in culture. To observe spines, neurons were transfected with pCAG-GFP at 7 days post-plating and fixed at 20 days. Mushroom spines in apical dendrites were reduced by about 30% in neurons from *Jnk1-/-* mice (Figure 3a), whereas there was an equivalent increase in thin spines *(*Figure 3b). Mushroom spines in basal dendrites also decreased, but this time there was no compensatory increase in thin spines, and the overall spine density was significantly lower in basal dendrites from cortical neurons lacking Jnk1 (Figure 3c–d). There was also an interesting increase in stubby spines in these neurons (Figure 3).

### 3.4. JNK Substrates DCX and MARCKSL1 Significantly Altered Dendritic Spine Maturation

**Figure 4:**
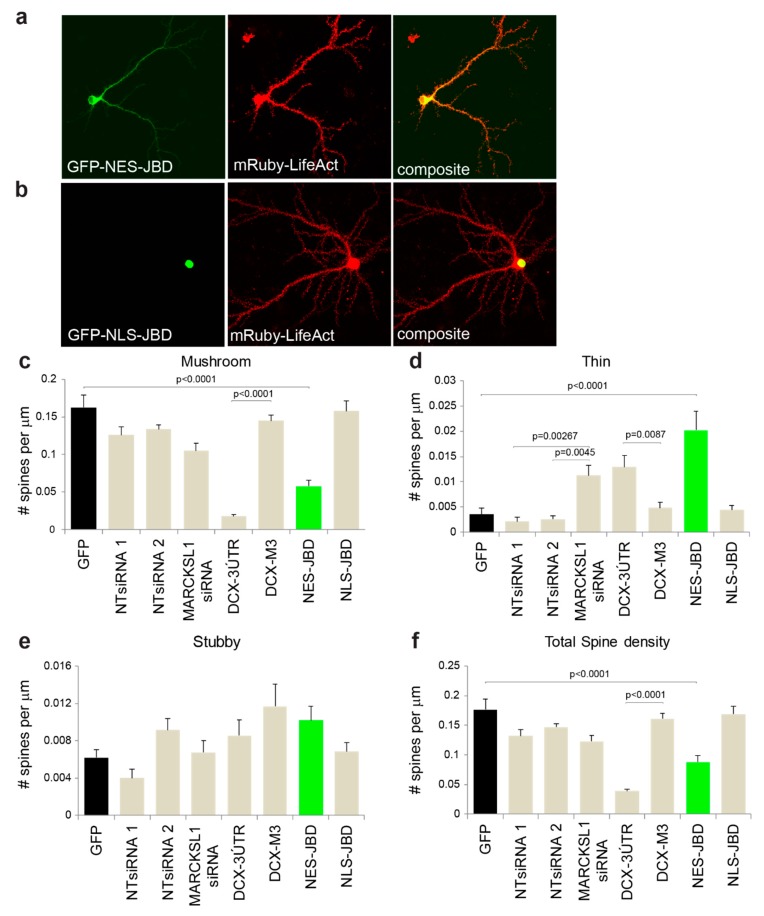
Inhibition of cytoplasmic JNK decreased mushroom spines and increased thin spines. (**a**,**b**) Representative images of GFP-NES-JBD (green for highlight purpose only) or GFP-NLS-JBD were co-expressed with mRuby-Lifeact in hippocampal neurons and fixed and imaged at 20 days post-plating. (**c**–**f**) Hippocampal neurons were transfected with EGFP ± non-targeting siRNA, siRNAs targeting myristoylated alanine-rich C kinase substrate-like protein-1 (MARCKSL1), shRNA targeting doublecortin (DCX), or control siRNA (DCX-M3). Spine morphology was measured in 21 day neurons. Inhibition of cytosolic JNK with EGFP-NES-JBD substantially reduced mushroom spine density while increasing thin spines. Similarly, knockdown of DCX substantially inhibited mushroom spine growth and increased thin spines. Significance was determined using one-way ANOVA followed by Sidak multiple comparisons test. Adjusted *p*-values are shown relative to GFP (for JBDs) or respective non-targeting (NT) siRNA or shRNA.

To address cell autonomous effects of JNK on hippocampal neuron spines and to test which substrates may be involved, we switched to hippocampal cultures. We first tested whether cytosolic or nuclear JNK activity was responsible for JNK regulation of spine density. To this end, we used compartment-targeted peptide inhibitors of JNK pEGFP-NES-JBD or pEGFP-NLS-JBD as previously described [26,27] (Figure 4a,b), where JBD stands for the “JNK binding domain” of JIP1a(1–277) that binds tightly to the common docking domain of JNK, thereby inhibiting substrate binding [26,45,46]. We found that expression of EGFP-NLS-JBD had no effect on dendritic spines compared to neurons expressing EGFP (Figure 4). However, expression of EGFP-NES-JBD significantly reduced the mushroom spine density while increasing the thin spines in dendrites of hippocampal neurons (Figure 4c–e). The same results were found in both basal and apical spines. Moreover, total spine density decreased upon inhibition of cytosolic JNK (Figure 4f).

We next selected the cytoplasmic targets of JNK that regulate actin, DCX, and MARCKSL1 [29,47], and tested whether they regulated spine morphology and density. Hippocampal neurons were transfected with shRNA targeting *Dcx* (DCX-3′UTR) or a mutant variant (DCX-M3) that does not silence *Dcx* [38]. *Dcx* knockdown decreased the number of mushroom spines by ≈80%, whereas thin spine number increased (Figure 4c–d). Expression of the mutant variant had no effect on spine number. Overall, spine density decreased upon *Dcx* knockdown, indicating that DCX played a requisite role in spine growth in hippocampal neurons (Figure 4f). We also tested whether or not MARCKSL1 expression was required. Using two independent siRNAs, we found that MARCKSL1 knockdown increased thin spines but had no significant effect on other spine types (Figure 4c–d).

### 3.5. Overexpression of DCX-T331D/S334D or MARCKSL1-S120D/T148D/T183D Differentially Rescued Spine Defects in Apical Dendrites of JNK-Deficient Neurons

**Figure 5:**
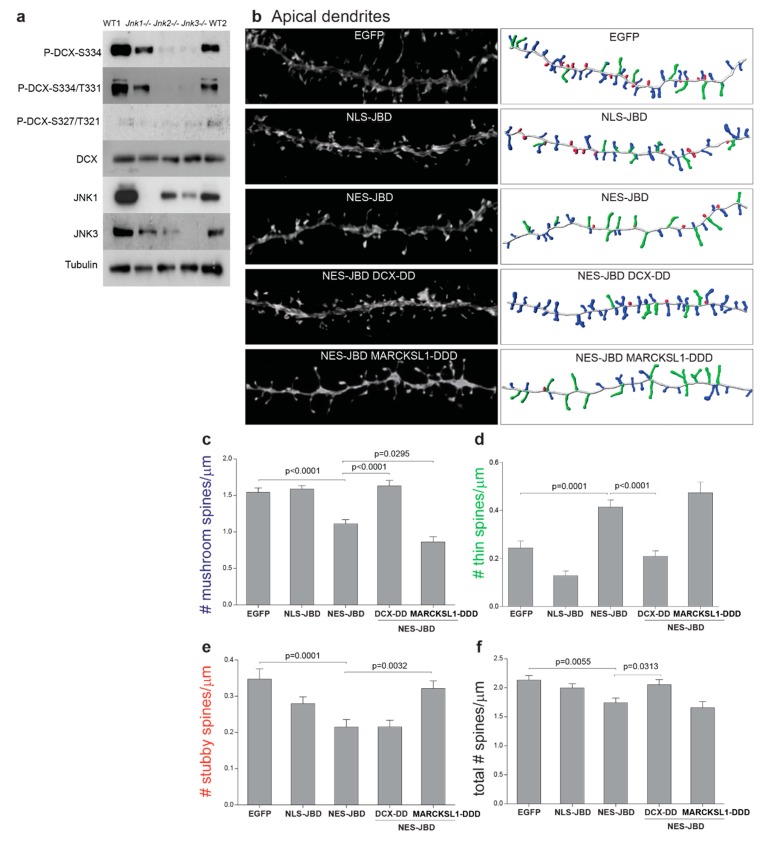
EGFP-DCX-T331D/S334D expression rescued mushroom and thin spines in apical dendrites of neurons expressing EGFP-NES-JBD. (**a**) Extracts from wild-type (WT1, WT2), *Jnk1-/-, Jnk2-/-*, or *Jnk3-/-* adult brains were immunoblotted using antibodies recognising phospho-DCX (P-DCX), DCX, JNK isoforms, or tubulin, as indicated. (**b**) Representative images (left) of hippocampal neurons expressing EGFP-NES-JBD, EGFP-NLS-JBD, EGFP-DCX-T331D/S334D (DCX-DD), or EGFP-MARCKSL1-S120D/T148D/T183D (MARCKSL1-DDD) are shown. 3D reconstructions of spines with spine type colour-coded for mushroom (blue), thin (green), or stubby (red) are on the right-hand side. (**c**–**f**) Once more, expression of EGFP-NES-JBD decreased mushroom spine density and increased thin spine density in apical dendrites. Normal spine density was recovered upon co-expression of EGFP-DCX-DD together with EGFP-NES-JBD. Spines were measured from between 15 and 22 apical dendrites from 6–10 cells per condition. Significance was determined using one-way ANOVA followed by Sidak Multiple Comparisons test.

We postulated that if DCX and/or MARCKSL1 were important mediators of cytosolic JNK in regulating spine development, overexpression of phosphomimicry mutants would rescue spine defects in neurons expressing GFP-NES-JBD. We first re-evaluated the JNK phosphorylation sites on DCX by phospho-blotting extracts from wild-type and *Jnk* knockout mouse brains (Figure 5a). This data validated the previous finding that GFP-DCX-T331 and -S334 were phosphorylated by JNKs [25,28], as phosphorylation on these sites was substantially depleted in *Jnk1-/-, Jnk2-/-*, and *Jnk3-/-* brains (Figure 5a). We next tested whether phospho-mutants would rescue spine development. We transfected hippocampal neurons at 7 days post-plating with EGFP-NES-JBD in the presence or absence of phosphomimicry mutants EGFP-DCX-T331D/S334D or EGFP-MARCKSL1-S120D/T148D/T183D. Cells were fixed at 21 days post-plating and dendritic spine density was analysed from 3D confocal images using Neurolucida software.

Expression of EGFP-DCX-T331D/S334D recovered normal numbers of mushroom and thin spines in neurons where cytoplasmic JNK was inhibited using EGFP-NES-JBD (Figure 5). Normal spine density was also rescued by EGFP-DCX-T331D/S334D (Figure 5b,f). However, neurons expressing EGFP-DCX-T331D/S334D did not rescue the loss of stubby spines (Figure 5b,e). In contrast, EGFP-MARCKSL1-S120D/T148D/T183D rescued stubby spines, recovering numbers to control levels (Figure 5b,e). These data showed that phosphomimicry mutants of DCX or MARCKSL1 on sites that JNK phosphorylates led to differential effects on spine morphology, where DCX-T331D/S334D played a more overt role, rescuing mushroom and thin spines as well as overall density, whereas MARCKSL1-S120D/T148D/T183D overexpression was only associated with stubby spine rescue.

### 3.6. Overexpression of DCX-T331D/S334D or MARCKSL1-S120D/T148D/T183D Differentially Rescued Spine Defects in Basal Dendrites of JNK-Deficient Neurons

**Figure 6:**
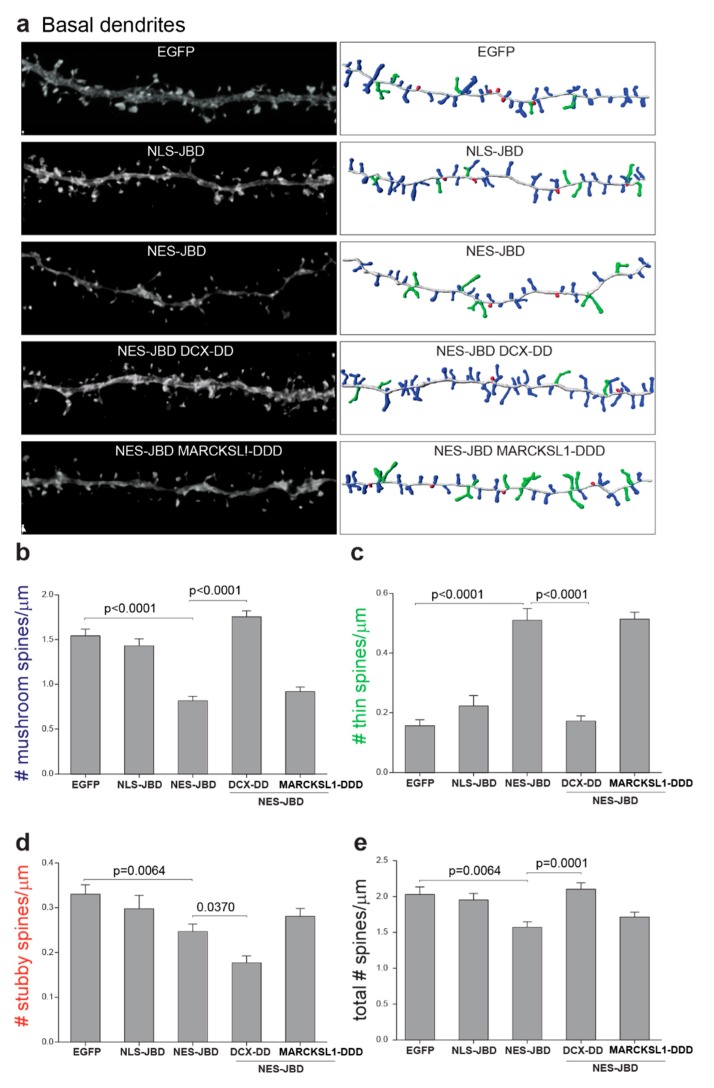
EGFP-DCX-T331D/S334D expression rescued mushroom and thin spines in basal dendrites of neurons expressing EGFP-NES-JBD. (**a**) Representative images of hippocampal neurons expressing EGFP-NES-JBD or EGFP-NLS-JBD together with mRuby2-Lifeact at 20 days post plating. Cells also expressed EGFP-DCX-DD or EGFP-MARCKSL1-DDD as indicated. (**b**–**e**) Additionally, in basal dendrites expression of EGFP-NES-JBD decreased mushroom spine density and increased thin spine density. Normal spine density was recovered upon co-expression of EGFP-DCX-DD together with EGFP-NES-JBD. Spines were measured from between 17 and 26 basal dendrites from eight cells per condition. Significance was determined using one-way ANOVA followed by Sidak multiple comparisons test.

In basal dendrites, an identical pattern of rescue was observed. Expression of DCX-T331D/S334D rescued the loss of mushroom spines and returned thin spines to normal levels in cells expressing GFP-NES-JBD (Figure 6a–c). Expression of DCX-T331D/S334D also induced recovery of spine density in cells expressing EGFP-NES-JBD. In basal dendrites, expression of MARCKSL1-S120D/T148D/T183D rescued stubby spine density to control levels (Figure 6a–e).

These data highlight a direct link between cytoplasmic JNK signalling and dendritic spine density and maturation, and show that phosphorylation of DCX on JNK phosphorylation sites T331 and S334 appears to be important for mediating these changes.

## 4. Discussion

In this study, we examined dendritic spine characteristics in *Jnk1-/-* mice using Lucifer yellow dye labelling. We found that mushroom spine density was decreased and thin spine density was increased in pyramidal neuron apical dendrites in hippocampus CA3. No equivalent changes were found in basal dendrites of the same neurons or in CA1. We went on to measure spine characteristics in neurons isolated from the cortex of *Jnk1-/-* mice. Here, we found more extensive differences. There were fewer mushroom spines in apical and basal dendrites of *Jnk1-/-* neurons, and overall spine density was reduced by 30% in basal dendrites. Consistent with these changes in spine density, *Jnk1-/-* mice struggled to learn the navigation task in the training session of the Morris water maze, traveling a greater distance to find the hidden platform.

Of note here, the changes to mushroom spines in hippocampus CA3 were restricted to pyramidal neuron apical dendrites. Specifically, the changes measured were on proximal dendrites that receive input from mossy fibres of dentate gyrus granule cells. Synaptic density changes in this region are expected to exert a large effect on neuronal activation due to proximity to the soma [48]. The specificity of this spine regulation in vivo may relate to the increased neurogenesis observed in the dentate gyrus of *Jnk1-/-* mice [49,50,51]. New-born immature granule cells, and the mature granule cells that they control, project directly via mossy fibres to apical dendrites in CA3 [52]. Therefore, decreased spine density observed in CA3 pyramidal neurons in vivo may result from altered innervation from the neurogenic niche. Although this is one possibility that remains to be tested, it is nonetheless clear from our experiments here in cultured cortical and hippocampal neurons that JNK can control spine morphology in a cell autonomous manner, and this does not distinguish between apical and basal dendrites. It is also notable that we did not observe changes in CA1 of *Jnk1-/-* mice, although only proximal dendrites were measured. Even still, the learning deficit could be explained by the CA3 spine changes alone, as lesions in CA3 are sufficient to disrupt spatial memory without altering neural activity in CA1 [53].

c-Jun N-terminal kinases are known to phosphorylate both nuclear and cytosolic targets. Among the nuclear targets, the transcription factor c-Jun is the most studied [54]. In Jun-S63A/S73A mice, where the JNK sites on c-Jun are mutated such that JNK cannot phosphorylate and transcriptionally activate it, long term potentiation is impaired [55], indicating a role for nuclear JNK in regulation of synaptic plasticity. This is, however, unlikely to be mediated by structural changes in spines per se, as Jun-AA mice exhibit no differences in spine morphology or density. Consistent with this, we observed no change in spine morphology or density when we inhibited nuclear JNK in hippocampal neurons. In contrast, we did observe 30% reduced mushroom and stubby spine density and increased thin spine density when we inhibited JNK in the cytoplasm. Thus, cytosolic targets of JNK must be involved in the regulation of hippocampal neuron spine maturation and density.

Among the cytoskeletal regulatory proteins that JNK phosphorylates is DCX [25,28]. Here, we demonstrated using gene knockdown that DCX was critical for maintaining mushroom spine density in hippocampal neurons, consistent with a previous study that showed the same in the olfactory bulb [56]. We also found that DCX suppressed filopodial number, as has been previously shown [47]. Importantly, here, we validated using brains from *Jnk1-/-*, *Jnk2-/-*, or *Jnk3-/-* mice that JNKs phosphorylate DCX on T331 and S334, and that JNK3 has the greatest impact. Finally, we demonstrated that expression of phosphomimicry DCX-T331D/S334D rescued normal mushroom and thin spine homeostasis. In addition to binding and stabilizing microtubules [57], DCX also interacts with actin [58,59], and it may be that interaction with both is necessary for recovery of spine homeostasis by EGFP-DCX-T331D/S334D. At least stabilization of actin alone appears insufficient, as EGFP-MARCKSL1-S120D/T148D/T183D, which binds and stabilizes F-actin but not microtubules [29], failed to recover mushroom and thin spine homeostasis. In contrast, MARCKSL1-S120D/T148D/T183D expression actually increased thin spine density.

In summary, we showed that genetic disruption of *Jnk1* impaired learning of a spatial navigation task in mice, and this was accompanied by disrupted spine morphology in apical dendrites of the CA3 hippocampal subdomain. In cultured hippocampal neurons, we demonstrated that inhibition of cytosolic JNK altered spine homeostasis via a cell intrinsic effect, increasing thin spines and decreasing mushroom spines. This may be important during synaptogenesis and is relevant for understanding known functions of JNK in synaptic plasticity regulation, including stress-induced memory loss and neurodegenerative disorders [60,61,62].
